# Assessment of various dimensions of impulsivity and their expression in unipolar and bipolar affective disorder

**DOI:** 10.1192/j.eurpsy.2024.545

**Published:** 2024-08-27

**Authors:** M. Dmitrzak-Weglarz, K. Bilska, M. Skibińska, B. Narożna, P. Zakowicz, A. Rajewska-Rager, P. Kapelski, J. M. Pawlak

**Affiliations:** ^1^Department of Psychiatric Genetics; ^2^Department of Pulmonology, Poznan University of Medical Sciences, Poznan, Poland

## Abstract

**Introduction:**

Impulsivity is the tendency to take quick and rash actions without the ability to assess their consequences, resulting in an increased frequency of risky behaviors. In recent years, it has been indicated that impulsivity is a multidimensional construct with different ways of expression in various mental illnesses. Moreover, personality traits might predispose do different psychiatric diagnoses and impact its course.

**Objectives:**

Because differences in the manifestation of impulsivity can be observed at several levels (e.g., behavioral/motor, cognitive, attention, or emotionally related), we applied several tools to check whether they would allow for the differentiation of unipolar (UD) and bipolar (BD) affective disorders.

**Methods:**

The study used data from 282 patients with affective disorders and 95 healthy controls of both sexes. Among the patients, we distinguished a subgroup diagnosed with UD and BD. We included a homogeneous group of patients in euthymia state at the end of hospitalization due to the last depressive episode. The following tools were used: subdimension novelty seeking (NS) of The Temperament and Character Inventory (TCI) and The Barratt Impulsiveness Scale version 11 (BIS-11) to assess various dimensions of impulsivity. The Coping Orientation to Problems Experienced (COPE) was used to assess the strategy of coping with stress. Statistical analyses were performed in Statistica 13.3 StatSoft, Krakow, Poland.

**Results:**

We observed significant differences in BIS-11 dimensions such as motor (MI) (p=0.0006), nonplanning (NP) (p=0.0249), and the sum of impulsivity (p= 0.0095) between UD and BD patients. We found no significant differences in the intensity of impulsivity measured by the NS subdimension, regardless of the type of affective disorder. In the Spearman rank correlation analysis, the following correlations of novelty seeking were revealed (p>0.05):

NS with BIS-11 MI (r_s_=0.3877, p=0001), BIS-11 NP (r_s_=-0.2926, p=0042) and COPE-planning (r_s_=-0.2552, p=0191) dimensions. Moreover, a unique and strong correlation of NS with COPE - focus on and venting of emotions was revealed in BD patients (r_s_=0.5402, p=0.0461).

**Conclusions:**

The obtained correlation results confirm the multidimensional nature of impulsivity. The relationship between NS and the motor and nonplanning dimensions comes to the fore. Among the tests used, BIS-11 best differentiated unipolar and bipolar patients.

**Disclosure of Interest:**

None Declared

